# Burden of Cancer Among Syrian Refugees in Jordan

**DOI:** 10.1200/JGO.18.00132

**Published:** 2018-10-11

**Authors:** Asem Mansour, Amal Al-Omari, Iyad Sultan

**Affiliations:** **All authors:** King Hussein Cancer Center, Al-Jubeiha, Amman, Jordan.

## Abstract

The Syrian crisis, which started in 2011, has had a profound impact on the entire region. Jordan, with its limited resources, now has the second highest ratio of refugees to inhabitants in the world (89 to 1,000). The actual number of Syrians in Jordan is hotly contested: more than 630,776 refugees registered in November 2015 compared with 1,265,514 reported by the national census conducted at the same time. Although the numbers are slowly but steadily increasing, the number of patients with cancer who were registered by the Jordan Cancer Registry peaked in 2013 at 510 patients reported and subsequently slumped downward, which coincided with changes in funding as a result of the increasing strains on the Ministry of Health. Older individuals, women, and patients with advanced diseases were less likely to be registered. These findings overlap with data obtained from the authors’ own center registry. Using age- and sex-specific population-based incidence rates, we estimated that 869 Syrians are diagnosed with cancer in Jordan annually. Using diagnosis-specific cost records of the King Hussein Cancer Foundation, we estimated that the cost of their treatments is 15.6 million Jordan dinars (US$22.1 million).

Fatima and her family thought they had overcome the most difficult hardships imaginable after they abandoned their home in Syria and fled to Jordan in 2013 to seek asylum and leave behind the suffering and woes of war. Their family outside Syria were scattered among different countries, and they knew nothing about the fate of the home they left behind. Despite such tribulations, the days ahead concealed a more difficult ordeal, as Fatima's belly began to swell. She had lost appetite and her weight dropped markedly, among other harrowing symptoms. The cause of this newfound distress was later found and diagnosed as ovarian cancer. The diagnosis came as an unbearable shock for Fatima's parents. She was only 12 years old, and they had no financial means to cover her costly treatment. A glimpse of hope appeared when the King Hussein Cancer Center (KHCC) agreed to assist with Fatima's case and allocated donations from the Goodwill Fund to cover her treatment. The doctors reassured the parents that Fatima had a chance for survival, because she was among the lucky Syrian patients with cancer who were treated at KHCC. However, it is disheartening to admit that the same is not the case for many refugee patients with cancer who go untreated because of lack of funding, among other reasons.

The Syrian crisis, which began in 2011, has had a profound effect on the Middle Eastern region.^1^ With limited resources, and because the blindsiding nature of the conflict led the entire region to find themselves unprepared for a crisis of such a scale, surrounding countries were faced with an unprecedented set of problems. Despite its struggling economy, scarce water resources, and increasing security threats, Jordan has become host to the second highest number of refugees (89 per 1,000 inhabitants) in the world. The number of refugees in Jordan is contested: Although the United Nations High Commissioner for Refugees (UNHCR) had registered 630,776 Syrians as of November 2015,^2^ the Jordan National Census, conducted at the same time, reported 1,265,514 Syrians in the country. The gap between the two numbers is alarming, but the discrepancy could reflect that some Syrians had moved to Jordan before the crisis because of many varied factors, including political asylum, marriage, work, and poverty. In addition, many Syrian refugees who moved to Jordan after the crisis elected to avoid registration through the UNHCR and used relatives in Jordan to provide them with basic needs and financial support.

It is notable that more than half of Syrian refugee households in Jordan reported at least one family member with a noncommunicable disease (NCD) and that a significant minority of these families did not receive any care because of financial reasons.^3^ It is important to point out that many refugees suffer from neuropsychiatric disorders and depression, which are considered NCDs.^4,5^ Poor access to medications and advanced health care are among the challenges that face refugees with NCDs in Jordan,^6^ and, although the public sector contributes significantly to the care of refugees with illness, out-of-pocket payments are commonly needed to receive proper care or medications.^7^ Traditionally, the focus among refugee populations had been on infectious diseases and malnutrition. Therefore, one problem that went largely unaddressed in the aftermath of the Syrian crisis was that many refugees and displaced people had cancer, which requires extensive resources for treatment, including financial support and medical facilities.

Cancer can be over-represented in refugees, as families with cancer are more likely to migrate out of hostile regions to seek medical care, a pattern which was previously reported during the Iraqi crisis.^8^ Conversely, and similar to comparable situations, under-reporting of cancer is also expected, because refugees may have difficulty integrating into the public health systems of their host country.^9^ Patients with advanced disease who may need palliative care are more likely to have funding refused.^10^ These patients may be over-represented in refugee groups because of late diagnoses and may be missed in national registries because they die without proper diagnoses or treatments.

Using data obtained from the Jordan National Census and age-specific cancer incidence among Syrians approximated by Globocan,^11^ we estimated that 869 Syrians in Jordan had cancer (55% women, 7% children younger than age 15 years; Table 1). Data provided by the Jordan Cancer Registry through December 2015 reported 1,553 patients with Syrian nationality during the period of 2011 to 2015.^12^ The numbers of Syrian patients with cancer registered in Jordan Cancer Registry increased in the first 2 years and peaked in 2013, when 510 patients were reported (53% women, 5% children). The numbers decreased significantly in 2014 (n = 353) and 2015 (n = 331). In a comparison of the numbers of these last 2 years to those of 2013 (the year with the highest number of registered patients), there were fewer women with cancer (50% *v* 53%), fewer adult patients (92% *v* 95%), and fewer patients with late-stage diagnoses (25% *v* 35%). Conversely, the types of cancer diagnosed were similar through the years; breast cancer, colorectal cancer, lymphomas and leukemia, brain cancer, and lung cancer topped the list.

**Table 1:**
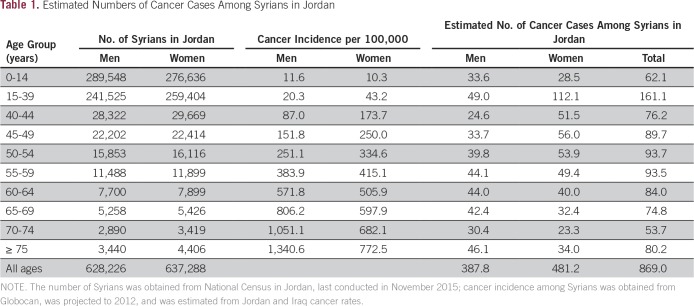
Estimated Numbers of Cancer Cases Among Syrians in Jordan

The difference in patient numbers across the years for which data are available is alarming and implies heavy amounts of under-reporting. The number of Syrian refugees who reside in Jordan has increased markedly since 2013, and the number of Syrian patients with cancer should have increased in parallel, comparable to the increase in the number of registered patients with cancer in the Kingdom. It is possible that women, older individuals with cancer, and those with advanced diseases are less likely to have access to medical care and hence are less likely to be reported in the collected data. Although international agencies and volunteer organizations provide various kinds of needed medical support, the expenses of treating refugee patients with cancer many times are not covered, because the cancers are designated as too poor of a prognosis and/or too financially costly to treat.^12^

The Government of Jordan had allowed Syrians registered with UNHCR to access health care services free of charge in Ministry of Health’s primary health care centers and hospitals as of March 5, 2012. However, in November 2014 this policy was withdrawn because of immense strains on the health care system, and Syrian refugees became required to pay the noninsured Jordanian rate when they used all types of health services provided by the Ministry of Health. This is a subsidized rate that is used for Jordanians who do not have government health insurance and is approximately 35% to 60% of what non-Jordanians (ie, foreigners) are paying. Though the noninsured Jordanian rate is normally affordable for nonvulnerable individuals, this change caused considerable hardship for many refugees and limited their access to health care, including cancer care provisions.^12^ Moreover, the UNHCR is facing serious limitations to support these patients and provide them with the treatment they need, because funding for humanitarian emergencies has been limited since the beginning of the Syrian crisis. Because of the limited resources and the overwhelming needs, the UNHCR selectively funds expensive treatments according to the decision of the UNHCR Exceptional Care Committee, which relies on several criteria to select the cases that most deserve to receive health care coverage. One such criteria is the aforementioned need of a disease prognosis.^10^

KHCC is a leading comprehensive cancer center in the region and is the only hospital dedicated entirely to cancer care in Jordan. KHCC provides cancer treatment to more than 4,000 new patients with cancer and 110,000 outpatients every year. During the period of 2011 to 2018, 356 Syrian patients have been treated at KHCC, and their treatment was covered by the King Hussein Cancer Foundation (KHCF) Goodwill Fund. The KHCF is an independent, nongovernmental, not-for-profit institution founded in 1997 by a Royal Decree to combat cancer in Jordan and the Middle East region. KHCF is the largest community-based organization in Jordan dedicated to combating cancer. KHCF work focuses on fundraising and development, global advocacy, public awareness about early detection and prevention, cancer coverage, and patient support. The cost of treating these 356 Syrian patients totaled 8,071,973 JOD (equivalent to approximately US$11,400,000). Using the cost of treatment per cancer type as provided by KHCF records and the expected number of patients with cancer diagnosed in 2017, we estimate that the annual cost of treating Syrian refugees is approximately 15,676,752 JOD (equivalent to US$22,111,118; Table 2), which is much larger than previous estimates^12^ and which can overwhelm the health care system of any country with limited resources.

**Table 2:**
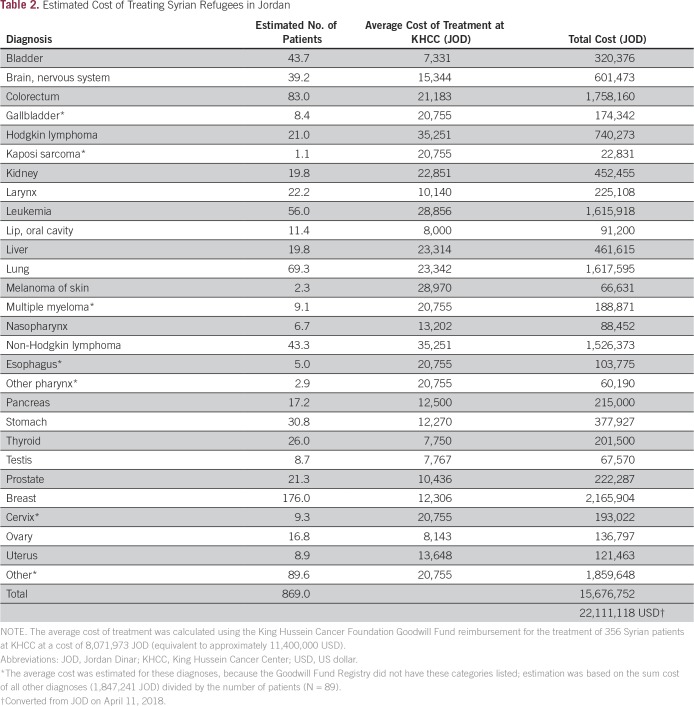
Estimated Cost of Treating Syrian Refugees in Jordan

The total number of Syrian patients registered in the KHCC hospital-based cancer registry during the period of 2011 to 2016 was 510 patients (compared with 13,908 Jordanians). The Goodwill Fund of the KHCF covered the treatment of almost two thirds of these patients. The percentage of pediatric patients was higher among Syrian patients (29%) than Jordanian patients (11%). This is properly caused by selection bias, because children with cancer were more likely to receive medical attention and funding. Women represented 46% of Syrian patients versus 54% of Jordanian patients. Notably, 20% of Syrian patients with breast cancer presented with distant metastasis compared with only 16% among Jordanians seen during the same period. This may support earlier reports that cancers reach a more advanced stage at the time of diagnosis among refugee patients with cancer^13^ and calls for screening and prevention initiatives for common cancers among refugees.^10^

Wars and conflicts can cause major delay in the diagnosis and treatment of displaced patients with cancer. In Jordan, cancer care for refugees is considered suboptimal because of limited financial coverage and access to care. Awareness and education for the prevention, screening, and early detection of cancer are also sorely lacking for refugee populations. Health care system capacities must be improved to absorb increasing demands, and financial support is crucial to cover the cost of treatment. The humanitarian community needs to consider the health care system of the country as a whole and not only for refugees to ensure harmony between the host community and refugees. Innovative and sustainable financing, such as social security and health insurance schemes, must be secured to ensure continuous coverage. Humanitarian responses should be coordinated with development organizations and donors, such as the World Bank, with an integrated approach that accounts for existing development plans and funds for the country and the added burden of a large refugee influx. A recommendation for patients to be treated according to resource-stratified guidelines rather than to receive suboptimal or no treatment could improve the sustainability of health care provisions and care for displaced patients with cancer. Financial coverage provided by the KHCF Goodwill Fund is helping treat some of the refugee Syrian patients with cancer; however, other nongovernmental and international relief organizations could play an important role in the support of refugees and displaced patients, and the international community is called upon to allocate more financial resources so at to alleviate the aching pain that these already suffering individuals are experiencing.
